# Dental Treatments During the COVID-19 Pandemic in Three Hospitals in Jordan: Retrospective Study

**DOI:** 10.2196/24371

**Published:** 2020-12-29

**Authors:** Lina Obeidat, Nader Masarwa, Amjad AlWarawreh, Waddah El-Naji

**Affiliations:** 1 Dental Department Royal Medical Services of Jordan Armed Forces Amman Jordan

**Keywords:** COVID-19, dental treatments, Jordan, lockdown, pandemic

## Abstract

**Background:**

Cases of COVID-19 first emerged in December 2019. Since then, the virus has spread rapidly worldwide, with daily increases in the numbers of infections and deaths. COVID-19 spreads via airborne transmission, which renders dental treatment a potential source of virus transmission. Dental treatments require the use of handpieces, ultrasonic devices, or air–water syringes, which generate considerable amounts of aerosols. Jordan, being one of the affected countries, instituted preventive lockdown measures on March 17, 2020. Emergency dental treatments were only allowed in dental clinics of the Royal Medical Services of Jordan Armed Forces and Ministry of Health, and were prohibited in other sectors such as private clinics and universities.

**Objective:**

The aim of this study is to investigate the dental treatments performed in three military hospitals during the 44-day lockdown period in Jordan. The investigation explores the impact of COVID-19 on the number of patients and types of performed dental treatments.

**Methods:**

Data such as number of patients, patients’ age and gender, and performed dental treatments were collected retrospectively from the hospital records and were analyzed.

**Results:**

Our results showed a 90% (17,591 to 1689) decrease in patient visits during the lockdown period compared to regular days. The total number of treatments (n=1689) during the lockdown period varied between endodontic cases (n=877, 51.9%), extraction and other surgical cases (n=374, 22.1%), restorative cases (n=142, 8.4%), orthodontic treatments (n=4, 0.2%), and other procedures (n=292, 17.3%). The differences in gender and age group among all clinics were statistically significant (*P*<.001 and *P*=.02, respectively).

**Conclusions:**

The COVID-19 pandemic had a significant effect on the number of patients seeking dental treatments. It also affected the types of treatments performed. Endodontic treatment accounted for almost 50% of patient load during the lockdown compared to approximately 20% during regular days.

## Introduction

The World Health Organization declared the outbreak of COVID-19 a Public Health Emergency of International Concern on January 30, 2020. On March 11, 2020, the outbreak was declared a pandemic [1]. COVID-19 is caused by a novel coronavirus, which is suspected to have originated from an animal host, followed by human-to-human transmission. The symptoms of COVID-19 are mainly respiratory, including fever, body ache, dry cough, fatigue, chills, headache, sore throat, loss of appetite, and loss of smell [2]. In severe cases, the symptoms worsen to cause respiratory failure. It can also affect other organs, leading to multi-organ failure caused by acute myocardial injury, renal failure, liver injury, or sepsis [3].

Dentists are among the highest occupational risk categories for the transmission and contraction of COVID-19. Routine dental treatments that produce significant amounts of aerosols, composed of saliva, blood, and tissue fluids, are considered to be at high risk for the spread of the virus, as it can spread via airborne transmission. Such treatments include the use of a turbine handpiece, air–water syringes, and ultrasonic scalers. During dental treatment, aerosols from a person who is infected or an asymptomatic carrier can transmit the virus directly to the dentist or dental assistant. Contact with contaminated instruments, surfaces, or airborne particles from such individuals is considered as the possible route of transmission [4]. Accordingly, the American Dental Association (ADA), National Health Service of the United Kingdom, and National Health Commission of China, along with other dental associations worldwide, urged dentists to postpone elective dental procedures and provide only emergency dental treatments [5-7]. The ADA has defined dental emergencies as “potentially life-threatening conditions that require immediate treatment to stop ongoing tissue bleeding and/or alleviate severe pain and/or infection including trauma, cellulitis, and uncontrolled bleeding” [8].

Jordan responded to the pandemic by implementing early lockdown from March 17, 2020 [9], followed by the declaration of a state of emergency on March 20, 2020, and then by implementation of a curfew. During the lockdown period, schools and universities were closed, public gatherings were banned, and borders and airports were shut down. Many activities and practices including public transport, hotels, and restaurants were also restricted. Among medical practices, dental clinics were closed, and emergency dental treatments were restricted to a few clinics in military hospitals and the Ministry of Health. Substantial protective measures were implemented in functional dental clinics to prevent cross-infection and the spread of the virus.

There is a need for establishing clear guidelines and regulations for the management of dental emergency procedures during possible future epidemic or pandemic situations. Accordingly, the aim of this study is to assess the dental treatments performed in three military hospitals during the lockdown period in Jordan. This research explores the impact of COVID-19 on the number of patients and treatments performed.

## Methods

This retrospective study was approved by the ethical committee of Royal Medical Services of Jordan Armed Forces. Data pertaining to patients requiring dental treatments during the lockdown due to the COVID-19 pandemic were obtained from the records of three major military hospitals in Jordan.

Data were collected from the lockdown and prelockdown periods. The lockdown period extended from March 17, 2020, to April 29, 2020 (44 days), during which the Government of Jordan had announced a total lockdown due to the COVID-19 pandemic; this period was referred to as T1. The prelockdown period extended from January 16, 2020, to February 29, 2020 (44 days), before Jordan recorded its first COVID-19 positive case on March 2, and this period was referred as T2. Data from T1 included the number of patients, their age and gender, and the performed dental treatments. Data from T2 included the number of patients and the performed dental treatments. The number of patients and performed treatments were compared between T1 and T2.

Data from T1 were entered and coded using the SPSS software version 17.0 (SPSS Inc). Values were reported as frequencies, means, and SDs. Cross-tabulation was used to test the correlations between variables. *P* values <.05 were considered statistically significant.

## Results

During T1, 1689 patients, with an average age of 35.04 (SD 10.96, range 14-87) years, were treated in the three selected major military hospitals. A total of 39 (2.3%) patients were older than 60 years, 650 (38.5%) were aged between 14 and 30 years, and 1000 (59.2%) were aged between 30 and 60 years.

Statistical analysis of the distribution of the 1689 patients visiting the dental clinics in T1 showed that 877 (51.9%) patients were treated in endodontic clinics and only 4 (0.2%) were treated in orthodontic clinics, as depicted in Table 1.

**Table 1:** Distribution of patients visiting the dental clinics during T1 (the lockdown period extending from March 17 to April 29, 2020; 44 days).

Clinics	Patients (n=1689), n (%)	Valid percentage (%)
Oral surgery	374 (22.1)	22.1
Endodontics	877 (51.9)	51.9
Restorative dentistry	142 (8.4)	8.4
Orthodontics	4 (0.2)	0.2
Others	292 (17.3)	17.3

Further analysis of the distribution of patients visiting dental clinics with respect to gender and age showed that, of all 1689 patients, almost two-thirds were male (n=1105, 65.4%) and one-third were female (n=584, 34.6%). The differences in gender and age groups among all clinics were statistically significant (*P*<.001 and *P*=.02, respectively), as shown in Table 2.

**Table 2:** Distribution of patients in terms of gender and age in different clinics during T1 (the lockdown period extending from March 17 to April 29, 2020; 44 days).^a^

Clinics	Male age groups (years), n				Female age groups (years), n			
	14-29	30-60	>60	Total	14-29	30-60	>60	Total
Oral surgery	86	113	5	204	62	106	2	170
Endodontics	252	371	14	637	82	151	7	240
Restorative dentistry	33	74	6	113	8	20	1	29
Orthodontics	1	0	0	1	3	0	0	3
Others	72	76	2	150	51	89	2	142
Total	444	634	27	1105	206	366	12	584

^a^The differences in gender (*P*<.001) and age groups (*P*=.02) were statistically significant.

The total number of patients had decreased by 90.4% (17,591 to 1689) when the number of patients visiting dental clinics was compared between T1 and T2. The highest reduction in the number of patients was recorded in orthodontic clinics, and the lowest reduction was observed in endodontic clinics (Table 3).

**Table 3:** Number of patients visiting dental clinics in T2 and T1.

Clinics	T2^a^, n	T1^b^, n	Change (%)
Oral surgery	2715	374	–86.2
Endodontics	3683	877	–76.2
Restorative dentistry	3018	142	–95.3
Orthodontics	6146	4	–99.9
Others	2029	292	–85.6
Total	17,591	1689	–90.4

^a^T2: prelockdown period extending from January 16, 2020, to February 29, 2020 (44 days).

^b^T1: lockdown period extending from March 17, 2020, to April 29, 2020 (44 days).

**Figure 1:**
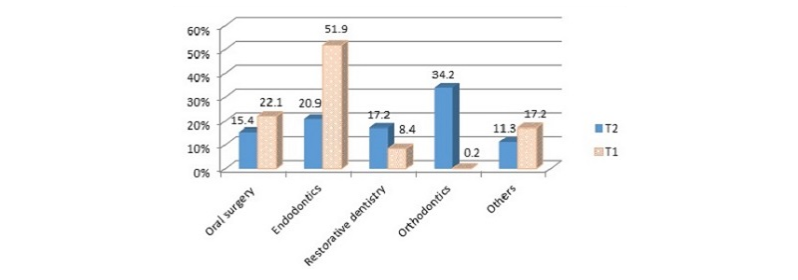
Distribution of treated patients in the dental clinics at T1 and T2. T1: lockdown period extending from March 17, 2020, to April 29, 2020 (44 days); T2: prelockdown period extending from January 16, 2020, to February 29, 2020 (44 days).

Although the total number of patients visiting dental clinics decreased in T1, there was a noticeable increase in the proportion of patients visiting endodontic and oral surgery clinics. Out of 1689 patients, 877 (51.9%) required endodontic treatment in T1, while out of 17,591 patients, only 3683 (20.9%) required treatment in T2. Out of 1689 patients, 374 (22.1%) were treated in oral surgery clinics in T1, while out of 17,591 patients, only 2715 (15.4%) were treated in T2 (Figure 1).

## Discussion

### Principal Findings

To the best of our knowledge, this is the first study to analyze the effect of the pandemic on dental treatments during the lockdown period in Jordan. In accordance with the results of other similar studies [10,11], the results of this study also confirmed that the COVID-19 pandemic had detrimental effects on the number of patients seeking dental treatments. Our results show that the COVID-19 pandemic even affected the distribution of patients in different dental specialties. During the lockdown period of 44 days, the number of patients who were treated in the three selected military hospitals was less than during the prelockdown period, in spite of the same duration of the periods (44 days). However, an overall decrease of approximately 90.4% (17,591 to 1689) in the number of patients visiting the dental clinics was observed, although the workload in the military hospitals was expected to increase to compensate for the obligatory closure imposed by the Government of Jordan on private dental practices and clinics across universities. Dental treatments were restricted to limited number of clinics in the Royal Medical Services and Ministry of Health during the lockdown period.

Several reasons could be attributed to the decrease in the number of dental patients and treatments. The primary reason could be the knowledge pertaining to the nature of COVID-19 spreading easily through aerosols, splashes, and droplets, inevitable with almost all types of dental treatments [12-14]. This knowledge has caused fear among patients regarding the possible transmission of the virus through dental treatments. Another reason that could have affected the number of dental patients in Jordan directly was the measures enforced by the Government of Jordan during lockdown; these measures included the ban on the use of private cars and public transport, and emergency transport of citizens being limited to Civil Defence Services [9].

There was also a decrease in the number of treatments performed in different specialty clinics, with the highest decrease being observed in the number of patients visiting orthodontic clinics. In total, only 0.2% (4/1689) of orthodontic patients visited the orthodontic clinics during T1 compared to 34% (6146/17,591) in T2. This represents a decrease of 99.9%, which can be explained by the fact that orthodontic emergencies are well tolerated. The number of performed restorations (amalgam, composite, glass-ionomer, and temporary fillings) also showed a 95.3% (3018 to 142) decrease during the COVID-19 lockdown.

This study showed that more male patients sought dental treatments than female. Of the 1689 patients, 1105 (65.4%) male patients sought dental treatments, in comparison to only 584 (34.6%) female patients. This result is in concordance with that of a similar study [15], which attributed the gender difference to the fact that women are more apprehensive toward dental treatment than men considering the possibility of respiratory infections. However, another study did not show any obvious difference between the number of male and female patients [10].

This total reduction in the number of patients treated in dentistry is alarming, as it increases the risk of dental health deterioration. The reluctance to seek treatment resulting from fear of the virus should not be underestimated. Understanding the current situation can help in the accurate prediction of future dental needs. In addition, requirements for dental services might increase dramatically post COVID-19.

The results of this study show that COVID-19 affected the distribution of patients in different dental specialties. A high percentage of treatments (877/1689, 51.9%) were performed for pulp-related pathosis such as acute pulpitis, acute apical periodontitis, and acute apical abscess. Endodontic emergencies account for the majority of dental emergencies in normal conditions [16], and in this study as well, endodontic emergencies contributed to the largest number of performed dental treatments.

A higher percentage of patients visited oral surgery clinics during the lockdown (374/1689, 22.1%) compared to the prelockdown period (2715/17,591, 15.4%). The performance of other procedures (examination, diagnosis, consultation, and referrals) increased in the COVID-19 lockdown (292/1689, 17.2%) compared to the prelockdown period (2092/17,591, 11.3%). This increase could be attributed to the fact that dentists chose to perform procedures with minimal aerosol generation to relieve the pain of patients, since health authorities worldwide had classified general dentists and dental hygienists as high-risk professions [17]. This has led to the development of fear among dentists and dental assistants regarding the possible transmission of the virus during the performance of dental procedures.

This study shows the effect of the COVID-19 pandemic on dental treatments performed during the lockdown period in Jordan; data from the prelockdown period served as a control. Additional studies are needed to analyze the effects of the COVID-19 pandemic on dental treatments performed in the postlockdown period. Jordan has not attained the peak of infection yet; therefore, the impact on dentistry should be analyzed while the COVID-19 cases are rising.

### Conclusion

The COVID-19 pandemic has had a substantial influence not only on the number of patients seeking dental treatments but also on the type of treatment performed. The overall decrease in the number of treated patients was 90.4% (17,591 to 1689). This decrease affected all dental specialties, especially orthodontics. However, endodontic treatments dominated the number of performed treatments during the COVID-19 lockdown, as 51.9% (877/1689) of performed treatments were related to endodontics.

### Recommendations

As the pandemic is still not under control, only focusing on the direct causes and control measures of COVID-19 alone could be shortsighted. The possible deterioration in the dental health of the population should be considered. Requirements for dental services might increase dramatically post COVID-19, especially in the field of orthodontics.

In case of a possible second lockdown, augmenting the endodontic specialty with adequate staff and more clinics to help in catering to the increased demands seems essential. Sufficient planning to organize and direct the available dental resources during and after the COVID-19 pandemic is the needed. There is also a need for regulations and preventive approaches in dental treatments to control the spread of COVID-19 in both governmental and private sectors.

## Abbreviations

ADA: American Dental Association
